# Rimmed Vacuoles in Becker Muscular Dystrophy Have Similar Features with Inclusion Myopathies

**DOI:** 10.1371/journal.pone.0052002

**Published:** 2012-12-14

**Authors:** Kazunari Momma, Satoru Noguchi, May Christine V. Malicdan, Yukiko K. Hayashi, Narihiro Minami, Keiko Kamakura, Ikuya Nonaka, Ichizo Nishino

**Affiliations:** 1 Department of Neuromuscular Research, National Institute of Neuroscience, National Center of Neurology and Psychiatry, Tokyo, Japan; 2 Department of Neurology, National Defense Medical College, Saitama, Japan; University of Minnesota, United States of America

## Abstract

Rimmed vacuoles in myofibers are thought to be due to the accumulation of autophagic vacuoles, and can be characteristic in certain myopathies with protein inclusions in myofibers. In this study, we performed a detailed clinical, molecular, and pathological characterization of Becker muscular dystrophy patients who have rimmed vacuoles in muscles. Among 65 Becker muscular dystrophy patients, we identified 12 patients who have rimmed vacuoles and 11 patients who have deletions in exons 45–48 in *DMD* gene. All patients having rimmed vacuoles showed milder clinical features compared to those without rimmed vacuoles. Interestingly, the rimmed vacuoles in Becker muscular dystrophy muscles seem to represent autophagic vacuoles and are also associated with polyubiquitinated protein aggregates. These findings support the notion that rimmed vacuoles can appear in Becker muscular dystrophy, and may be related to the chronic changes in muscle pathology induced by certain mutations in the *DMD* gene.

## Introduction

Rimmed vacuoles (RVs) can be seen in a certain range of muscle diseases including distal myopathy with rimmed vacuoles (DMRV) and sporadic inclusion body myositis (sIBM), myofibrillar myopathies, and also lysosomal myopathies [1]–[3]. By lysosomal enzymatic activities and electron microscopic features, RVs represent accumulation of autophagic vacuoles [4]. RVs are thought to be due to lysosomal dysfunction or due to accumulation of the various proteins that affect progression of the autophagic process within myofibers [1]–[2], [5].

Becker muscular dystrophy (BMD) is a dystrophinopathy caused by mutations in *DMD* gene that shows a milder disease course as compared to Duchenne muscular dystrophy (DMD). BMD patients show a wide variety of symptoms from gait disturbance in early childhood to almost no weakness even in adulthood. Through our muscle repository, we noted that some dystrophinopathies also show RVs in the muscles, albeit rare [6]. Because dystrophinopathies are related to membrane fragility of myofibers, the presence of RVs in BMD patients is perplexing and raises several issues that need to be clarified, like the relevance of RVs in BMD and the frequency of BMD patients that show RVs in myofibers. The second issue is the clinical and pathological features of BMD muscles associated with RV formation. The third issue is the characters of the RVs in BMD in comparison with those seen in the other disorders.

In this study, we focused on BMD patients who showed RV in muscle biopsy sections, and noted genetic and clinical characteristics, in addition to features seen in muscle pathology. Extensive immunohistochemical analysis was performed to note the nature of these RVs in comparison to those seen in IBM.

## Materials and Methods

### Ethics Statement

This study was approved by the ethics committee in National Center for Neurology and Psychiatry, Japan. All data from patients were obtained through written informed consent.

### Patients

From the muscle repository of National Center for Neurology and Psychiatry, we identified patients having deletion and mutation in *DMD* gene. The clinical information of each patient was carefully reviewed, and the following data were included for analysis: age at onset of disease, age at biopsy, disease duration, and serum creatine kinase (CK) level. For control, we included samples from patients genetically diagnosed as DMRV (*n* = 2) or sIBM (*n* = 2).

### Histochemistry

All biopsied muscles were frozen in liquid nitrogen-cooled isopentane and kept at −80°C. Transverse serial sections of frozen muscles with 8 µm thickness were stained with H&E, modified Gomori trichrome (mGT) and a battery of histochemical methods, including acid phosphatase and nonspecific esterase [7].

For histological analysis, the following parameters were noted for the evaluation of specific pathological characters: number of necrotic and regenerating fibers (defined as homogeneously pink and basophilic fibers on H&E staining, respectively); fiber type composition as determined by ATPase staining with pre-incubation at pH 4.6 and pH 10.0; occurrence of RVs seen on mGT staining; number of atrophic fibers; and other characteristic pathology. All routine histochemistry and immunohistochemistry analysis were done on adjacent serial sections. Modified gomori stain was done before and after immunohistochemistry panel to ensure the presence of rimmed vacuoles in the slides. Microscopic observation was performed using OLYMPUS BX51 (Olympus).

### Genetic Analysis

Genomic DNA of patients was isolated from peripheral blood or muscle specimen using standard protocols. Multiplex ligation-dependent probe amplification (MLPA) or multiplex PCR method were done using standard protocols [8]. Genomic sequencing analysis of all the exons and flanking introns of the *DMD* gene was done in patients without deletion by MLPA. Sequence for primers used for *DMD* gene analysis are available upon request.

### Immunohistochemistry

We performed indirect immunofluorescence staining on 7-µm serial sections of muscle according to previously described methods [9], [10]. After immersion in a blocking solution, sections were then incubated at 37°C for 2 hours with primary antibodies against dystrophin (DYS-1, DYS-2 and DYS-3, 1∶500, 1∶50 and 1∶10) (Novocastra), sarcoglycans (SGCA, SGCB, SGCG, and SGCD, 1∶500, 1∶20, 1∶500 and 1∶20) (Novocastra), laminin-α2 chain (1∶50,000) (ALEXIS), α- and β-dystroglycan (1∶50 and 1∶100) (Upstate Biotech), dysferlin (1∶2,500) (Novocastra), emerin (1∶20) (Novocastra), collagen VI (1∶2,500) (Novocastra), HLA-ABC (1∶5,000) (DAKO), caveolin-3 (1∶200) (Transduction laboratories), lysosomal associated membrane protein 1 (LAMP-1) (1∶50) (DSHB), LC3 (1∶100) (Novus biologicals), amyloid precursor protein (APP) (1∶200) (Covance), beta-amyloid 1–42 (Aβ1-42) (1∶100) (Chemicon), polyubiquitin (polyUb) (1∶100) (Biomol), CD68 (KP1) (1∶100) (Dako) and p62/SQSTM1 (1∶200) (Biomol). After washing, slides were subsequently incubated at room temperature for 30 minutes with a secondary antibody, either Alexa-labeled donkey anti-mouse or anti-rabbit IgG (1∶600) (Invitrogen), or rhodamine-labeled goat anti-mouse IgM (1∶600) (TAGO), as appropriate. Sections were observed using KEYENCE BZ-9000 and digital images were analyzed by BZ-II Analyzer 1.03 (KEYENCE).

### Electron Microscopy

Biopsied muscles were fixed in buffered 2% isotonic glutaraldehyde at pH 7.4, post-fixed in osmium tetroxide, dehydrated, and then embedded in Epoxy resin, according to standard protocols [7]. Ultra-thin sections were stained with uranyl acetate and lead nitrate, and observed under a Tecnai Spirit Transmission Electron Microscope (FEI).

### Statistical Tests

For analyzing clinical information of BMD patients with RVs as compared to BMD patients without RVs, non-parametric Mann-Whitney test or unpaired *t* test with Welch correction were used as appropriate. A *P* value less than 0.05 was considered significant. Statistical analysis was performed using GraphPad Prism 5.03 (GraphPad Software).

## Results

Our patient cohort was composed of 65 patients diagnosed to have BMD as supported by *DMD* gene deletion (64/65) and mutation (1/65). Among these BMD patients, we identified 12 patients (18.5%) who had RVs in myofibers on muscle biopsy. By MLPA and multiplex PCR, 4 patients had in-frame deletions of exons 45–47, 5 patients had deletions of exons 45–48, one had deletion of exons 45–53, and one had deletion in exons 14–41. The remaining patient (Patient 12) was identified to be carrying a novel missense mutation (c.5827A>G, p.Met1943Val, in exon 41; Table 1) by direct sequencing of *DMD* gene. This mutation was not identified in 100 control chromosomes. We excluded the involvement of mutations in *GNE* gene, which is a causative gene for DMRV by Sanger sequencing (data not shown).

**Table 1:** Summary of clinical and pathological findings of BMD patients with rimmed vacuoles.

Patient No.	1	2	3	4	5	6	7	8	9	10	11	12
DMD exon deletion/point mutation	45–47	45–47	45–47	45–47	45–48	45–48	45–48	45–48	45–48	45–53	14–41	c.5827A>G p.Met1943Val
Clinical findings												
age at onset (years)	6	13	20	20	5	13	35	44	33	39	32	57
symptom at onset	cramp	weakness	weakness	weakness	hypertrophy	weakness	weakness	pain	weakness	weakness	weakness	weakness
age at biopsy (years)	34	35	41	43	22	41	49	54	74	45	34	60
serum CK activity (IU/L)	1233	3061	1076	1136	2788	981	481	1327	2420	830	2428	2424
Pathological findings												
biopsied muscle	BB	unknown	BB	BB	unknown	BB	QF	RF	BB	QF	QF	BF
fiber type population												
type 1 (%)	10	40	40	82	37	36	7	21	76	41	12	84
type 2A (%)	38	16	33	12	23	23	36	41	13	32	30	11
type 2B (%)	50	42	23	1	38	41	55	36	5	24	10	1
type 2C (%)	2	2	4	5	2	1	2	2	6	3	1	4
fibers with internally placed nuclei (%)	10	15	30	5	25	50	50	15	10	30	15	70
fibers with rimmed vacuole*	10	42	5	2	6	21	29	7	11	78	1	13
small atrophic fibers*	10	154	137	57	4	42	88	7	11	354	0	26
necrotic fibers*	0	0	5	0	0	2	0	3	4	3	2	0
regenerating fibers*	2	2	32	0	12	2	0	7	4	3	2	9

BB = biceps brachii; QF = quadriceps femoris; RF = rectus femoris; BF = biceps femoris; *per 1,000 fibers

We use the term “BMD with RV” to delineate the patients who had RVs from the “BMD without RV” patients who did not have RVs in muscle sections. Deletions of exons 45–47 and 45–48 in *DMD* gene were frequent in both of BMD patient groups and the frequency of these two mutations was 9 out of 12 patients (75%) in BMD with RV group and 18 out of 53 patients (35%) in BMD without RV group. We further analyzed clinical information of only patients with deletions of exons 45–47 and 45–48 (BMD with RV patients 1–9 in Table 1 and BMD without RV group in Table 2). In terms of demographic data, BMD with RV patients were slightly older at age of disease onset (21.0±4.5 years in BMD with RV versus 19.5±3.5 years in BMD without RV, *P* = 0.3), age at biopsy (43.7±4.9 years in BMD with RV versus 34.5±4.7 years in BMD without RV; *P*≤0.002) and slightly longer mean disease duration (22.7±3 years in BMD with RV versus 15.4±3.9 years in BMD without RV; *P* = 0.15). Serum CK levels in BMD with RV was slightly lower (1611±301 IU/L in BMD with RV versus 2605±964 IU/L in BMD without RV; *P* = 0.12).

**Table 2:** Summary of clinical and pathological findings of BMD patients without RVs (*DMD* deletion exons 45–47 and 45–48).

Patient No.	13	14	15	16	17	18	19	20	21	22	23	24	25	26	27	28	29	30	31
DMD Deletion	exons 45–47									exons 45–48									
Clinical findings																			
age at onset(years)	22	30	45	14	4	unknown	38	14	5	4	5	13	15	16	2	45	7	33	39
symptom at onset	weakness	weakness	weakness	weakness	atrophy	unknown	weakness	high CK	pain	pain	weakness	pain	weakness	weakness	cramp	weakness	weakness	weakness	weakness
age at biopsy(years)	24	46	60	48	19	23	48	14	6	14	10	53	58	64	16	48	10	56	40
serum CK activity(IU/L)	994	1487	942	569	1193	unknown	321	2705	15540	3044	11174	549	513	245	2702	758	2945	615	600
Pathological findings																			
biopsied muscle	QF	deltoid	BB	BB	QF	unknown	BB	GC	BB	BB	BB	BB, QF	BB	GC	QF	BB	BB	TA	GC
fiber typepopulation																			
type 1 (%)	10	66	36	42	16	54	70	28	46	41	38	50	67	47	27	40	48	80	54
type 2A (%)	44	28	47	49	67	23	22	59	32	24	55	26	28	31	51	38	45	20	30
type 2B (5)	46	3	16	10	9	22	4	11	20	33	4	21	6	13	12	17	4	0	16
type 2C (%)	0	3	1	0	0	2	4	2	2	2	3	3	0	8	10	5	3	1	1
small atrophicfibers*	73	32	0	278	56	62	115	3	11	16	7	115	0	78	150	51	24	6	80
necrotic fibers*	0	0	0	0	0	8	1	3	2	3	1	0	0	2	3	0	5	0	0
regeneratingfibers*	4	22	0	0	0	0	3	13	13	1	15	2	0	0	3	2	7	3	5

QF = quadriceps femoris; BB = biceps brachii; GC = gastrocnemius; TA = tibialis anterior; *per 1,000 fibers

**Figure 1:**
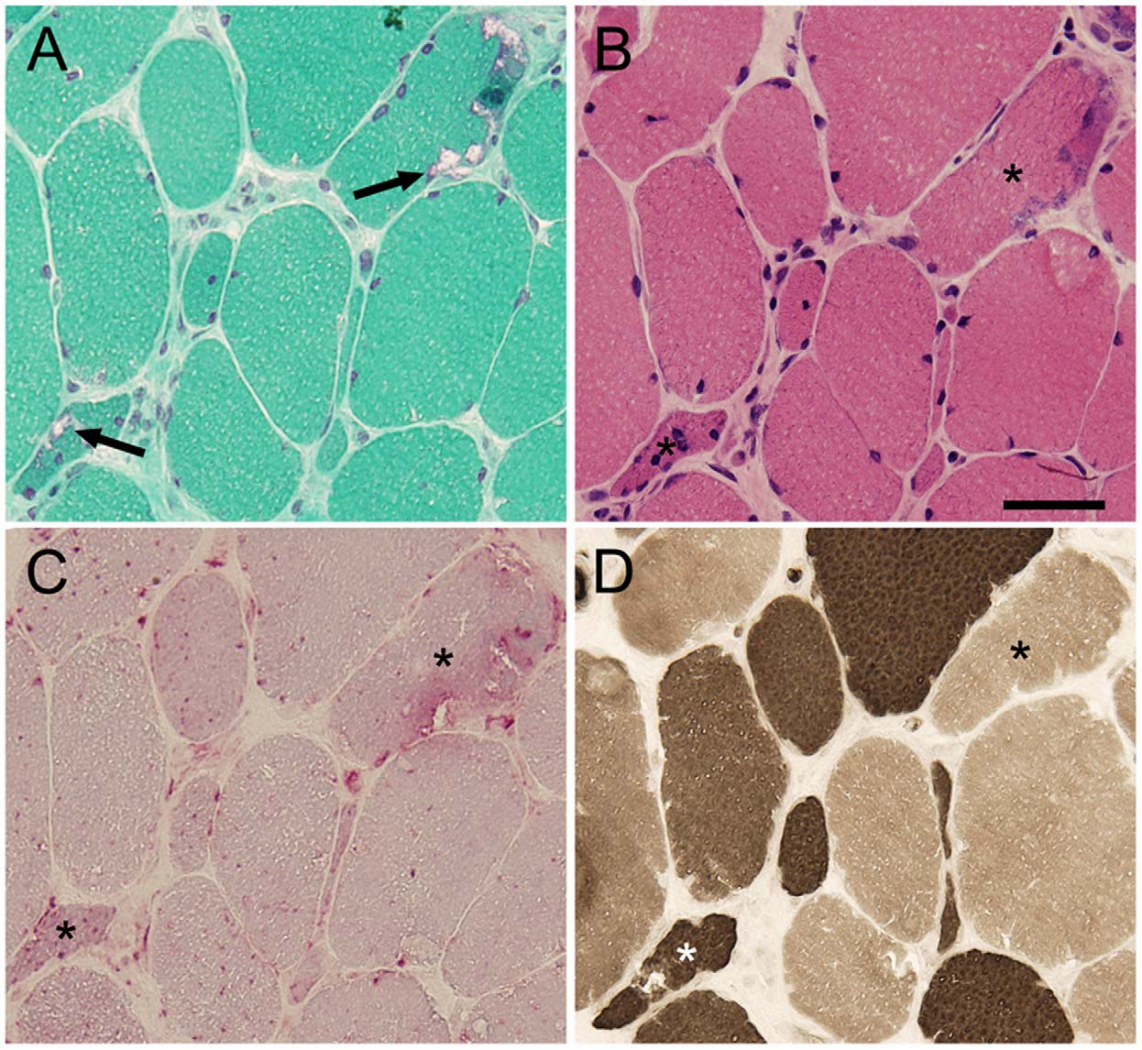
Pathological Characteristics of BMD patients. A: On mGT staining, RVs are seen in the periphery of myofibers (**arrow**). **B:** On H&E staining, there is marked variation in fiber size with scattered small atrophic fibers. **C:** High acid phosphatase activity is seen in the areas of RVs. **D:** On ATPase staining pre-incubated at pH 4.6, RVs are seen in both type 1 and type 2 fibers. **Asterisks** indicate myofibers with RVs. Scale bar: 25 µm.

With regards to muscle histochemistry, all RVs in BMD were highlighted on mGT staining (Figure 1A, arrow). In addition, BMD with RV patients revealed myopathic change with moderate variation in fiber size by the presence of scattered small atrophic and angular fibers, while necrotic and regenerating fibers are rare, on H&E staining (Figure 1B). Acid phosphatase staining confirmed strong lysosomal enzyme activities within fibers with RVs (asterisks in Figure 1C). On ATPase staining, RVs were seen in both type 1 and type 2 fibers (Figure 1D) and small atrophic fibers were not type 2C fibers. Positive correlation (R^2^ = 0.790) between number of fibers with RVs and that of small atrophic fibers is seen (data not shown), suggesting a close relationship on the occurrence of RVs and the presence of atrophic myofibers.

**Figure 2:**
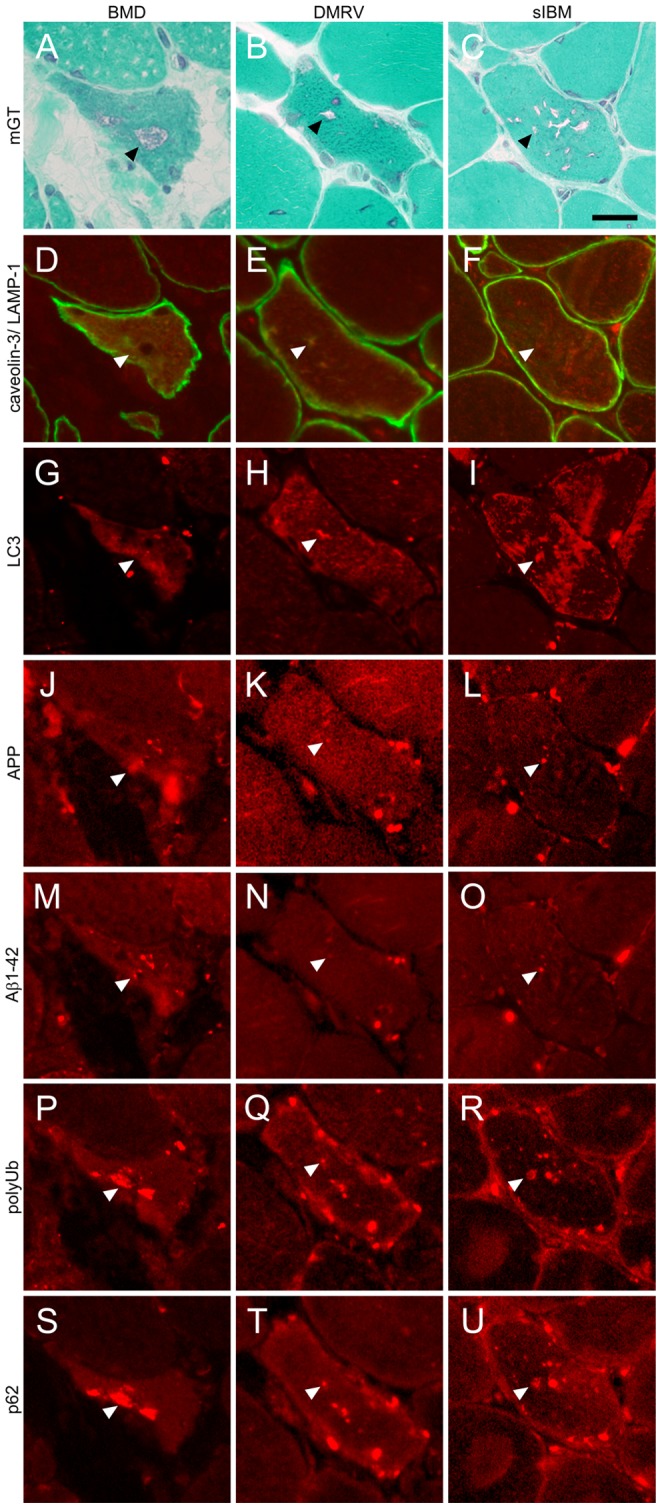
Immunohistochemical characteristics of RV in BMD compared to DMRV and sIBM. Representative transverse serial sections of biopsied skeletal muscles from BMD with RV (left column), DMRV (center column) and sIBM (right column) patients. **A–C:** mGT staining similarly highlights the fibers with RVs (**arrowheads**) in all patients. **D–F:** LAMP-1 (red) co-stained with caveolin-3 (green), **G–I:** LC3, **J–L:** APP, **M–O:** Aβ1-42, **P–R:** polyUb proteins, and **S–U:** p62. Immunofluorescent signals are observed around RVs (**arrowhead**). Scale bar: 25 µm.

**Figure 3:**
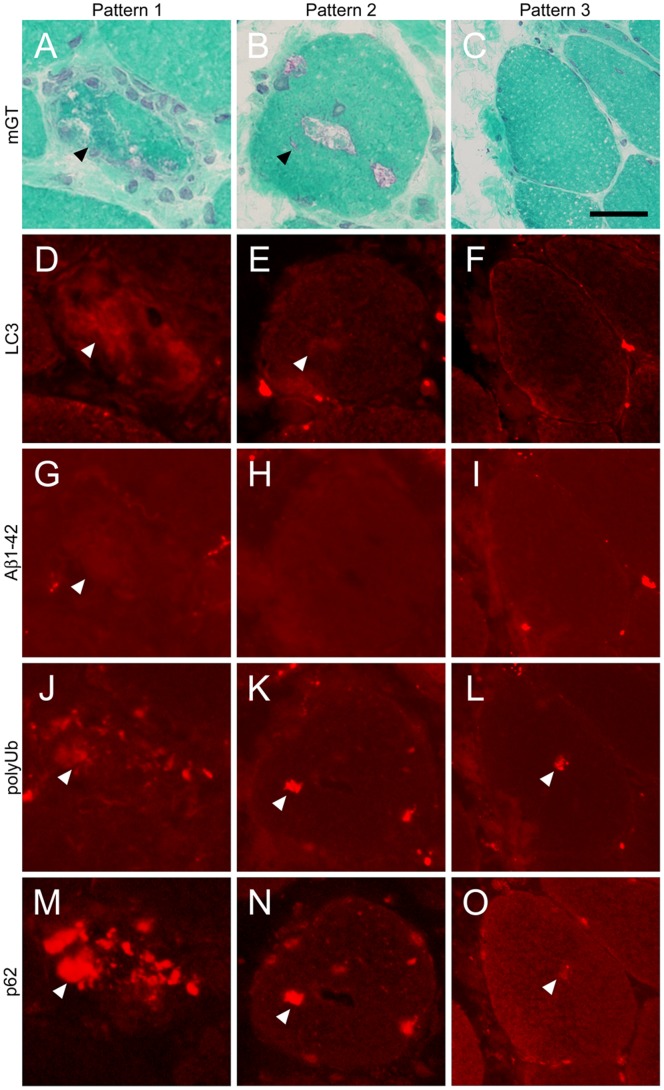
Patterns of immunohistochemical findings in BMD with RV. Representative transverse serial sections of biopsied skeletal muscles from BMD patients with RV. **A–C:** mGT shows the presence of RVs (**arrowheads**). **D–F:** LC3, **G–I:** Aβ1-42, **J–L:** polyUb, and **M–O:** p62. Immunofluorescent signals are seen within the fibers with RVs (**arrowheads**). Pattern 1 (left column) shows similar characteristic staining of RV fibers as DMRV and sIBM. Pattern 2 (center column) show almost similar characteristics as pattern 1, except for the faint staining of Aβ1-42. Pattern 3 (right column), with rare occurrence, shows myofibers with RVs that are negatively stained by LC3 and Aβ1-42. Scale bar: 25 µm.

To further characterize RVs in dystrophinopathy, we performed immunohistochemical analysis in BMD with RV patients (Figure 2, left column) in comparison with DMRV (Figure 2, center column) and sIBM (Figure 2, right column) patients; reference RVs are shown in mGT (Figure 2A–C). In RVs and areas in proximity, the lysosomal protein LAMP-1 (Figure 3D–F) and the autophagic vacuole marker LC3 (Figure 2G–I) were positively stained. As accumulation of several proteins is considered to be associated to RV formation in DMRV and sIBM [1], [11], [12], we observed the accumulation of APP (Figure 2J–L), Aβ1-42 (Figure 2M–O) and polyUb protein (Figure 2P–R) in and around RV in BMD with RV, DMRV and sIBM patients. We also tried to examine p62, which marks proteins for autophagic degradation in the sites with polyUb protein accumulation [13]. The staining pattern of p62 was similar to that of polyUb protein (Figure 2S–U). We also stained sections from BMD with RV, DMRV and sIBM patients with CD68 antibody, a macrophage marker, and Alexa-labeled anti-mouse IgG secondary antibody alone. Both staining were negative in RV positive fibers (Figure S1).

From our immunohistochemical analysis, we classified three patterns of staining. First, most myofibers with RV were immunoreactive to amyloid, polyUb proteins and p62 (Figure 3, left column; Pattern 1). Second, some fibers with RV showed negative for amyloid but positive for polyUb proteins and p62 (Figure 3, center column; Pattern 2). Interestingly, the third pattern consisted of some myofibers without RV that are positively stained only with polyUb proteins and p62 (Figure 3, right column; Pattern 3).

**Figure 4:**
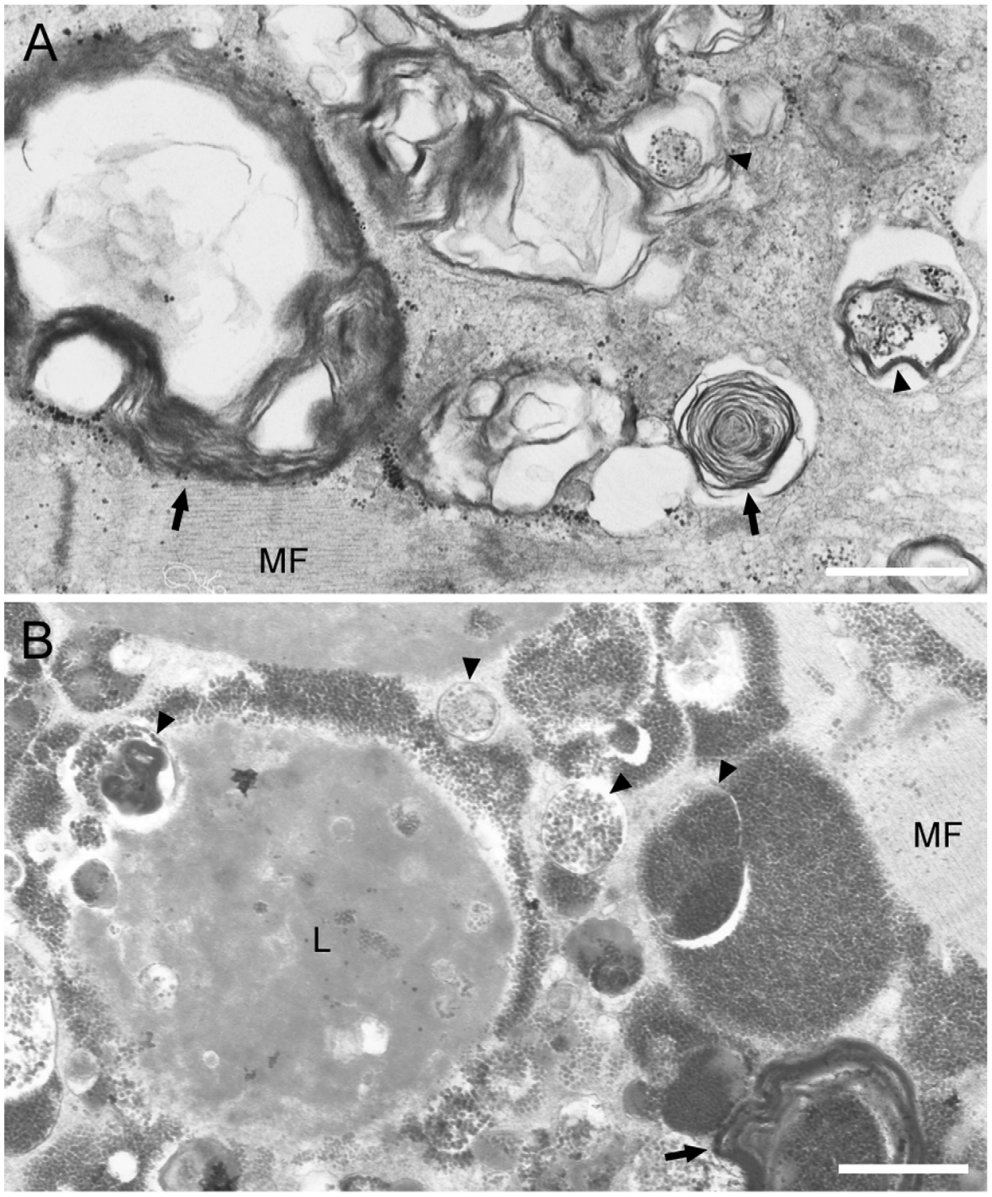
Areas of RVs in BMD myofibers show typical electron microscopic characteristics of autophagic vacuoles. A: Accumulation of autophagic vacuoles (**arrowheads**), various cellular debris, and multilamellar bodies (**arrow**) are seen in myofibers of some BMD patients. Note the intact arrangement of myofibrils (**MF**) surrounding autophagic area. B: In areas with or without autophagy, lipofuscin deposit (**L**) is seen. Scale bars: 1 µm.

To have a closer look at the structure of these fibers with RV, we performed electron microscopy and observed the presence of autophagic vacuoles and multilamellar bodies within myofibers. In the areas surrounding autophagic vacuoles (Figure 4A), however, myofibrillar structures are almost maintained except for partial distortion of Z-line. Furthermore, lipofuscin granules were also observed around autophagic vacuoles (Figure 4B). We also confirmed Nile blue staining and confirmed that lipofuscin granules were accumulated in the fibers of BMD patients with RVs (data not shown).

## Discussion

### RVs in BMD are Rare but may be Related to Certain Types of *DMD* Mutations that Cause Milder Phenotype

We found 12 patients who showed RVs in muscle pathology among 65 BMD patients, representing surprising rate of 18.5%. In DMD and BMD, a genotype-phenotype correlation has been established [14]–[17]. Deletion of exons 45–55, for example, has been reported to be associated with quite mild muscle weakness [18]–[19]. Interestingly, in a previous report, one BMD patient who showed RV in his skeletal muscle section had a deletion from exons 45–48 in the *DMD* gene and showed mild to moderate weakness in lower girdle muscles [6]. In our series, the deletions in exons 45–47 or 45–48 in *DMD* gene were frequently found in the patients with RV. Our BMD with RV patients also showed a mild course of disease, with later onset and mild elevation of CK, similar as previous reports on the patients with the same deletion on *DMD* gene [6]. Additionally, in spite of similar age of disease onset in the patients with and without RV with the deletions in exons 45–47 or 45–48, the higher mean biopsy age in the patients with RV may suggest that the milder clinical course and longer disease duration in dystrophinopathy could contributory for the formation of RV in muscle. It should be noted, however, that the similar clinical course, in age of onset and biopsy, and serum CK activity, can be found in some patients in both groups, BMD with or without RV, implying that RV formation may be one of the phenotypes in patients with such deletions, or one that belongs to the disease spectrum of a mild myopathic process.

### BMD Patients with RV Show Chronic Myopathic Features in Histology

Consistent with serum CK level, there were scattered necrotic and regenerating fibers observed in muscles of BMD patients without RV, while BMD patients with RV rarely show necrotic and regenerating or type 2c fibers. An increase in the number of small atrophic fibers in BMD patients with RV was remarkable as that in the patient who is previously reported [6]. This characteristic pathology is rather like myopathic changes as observed in other late-onset chronic myopathies [20].

### Lipofuscin Accumulation in BMD

The number of lipofuscin granules was strikingly higher in myofibers of BMD with RV patients. Lipofuscin pigments consist of proteins and lipid containing peroxidation products of polyunsaturated fatty acids, which are formed in relation to oxidative stress, and aging process. Lipofuscin granules are highly seen in postmitotic cells and also characterized as undigested inclusion of amyloid proteins and other proteins due to lysosomal dysfunction in aged and diseased muscle and in the central nervous system [21]–[22].

Several papers reported that oxidative stress is implicated as a pertinent factor involved in pathogenesis of dystrophin-mutated muscular dystrophies [23], [24]. In a severe DMD, dystrophin deficiency is proposed to cause profound oxidative damage, which may induce muscle necrosis that is thought to trigger the necrosis-regeneration necessary for renewal of myofibers. In contrast, in a milder BMD, although oxidative stress presumably is present at a lower level, it may lead to chronic accumulation of oxidized proteins and lipids in the absence of active necrosis and regeneration. We can only speculate that the myofibers in the BMD patients may mimic senescent status in which cellular homeostasis are slowed down. The presence of chronic myopathic changes, composed of myofiber inclusions, fiber atrophy and RV formation in BMD may reflect a long-standing process as exposed to oxidative state.

### Common Mechanism of RV Formation in BMD to those in DMRV and sIBM

We found that only polyUb proteins and p62 can be seen deposited even in some fibers without and with RV. These results imply that at first, polyUb proteins were accumulated and then they recruited p62 to induce selective autophagy, as observed in neurodegenerative diseases [25]. Although in this study, we did not identify the polyUb proteins, Henderson et al. reported that the internally deleted-dystrophin minigene constructs revealed no cooperative transition during thermal denaturation and significant protein aggregation, suggesting increased susceptibility to misfolding, instability and aggregation of internally deleted-dystrophin proteins [26]. Further experiments on the identified dystrophin mutants will be required to clarify this issue.

Despite induction of autophagic process by recruitment of p62, the polyUb proteins are degraded, then leading to accumulation of numerous numbers of autophagic vacuoles. Such findings of polyUb and p62 accumulation and numerous numbers of autophagic vacuoles and multilamellar bodies in myofibers (Figure 4A, B) are strikingly similar to those seen in a model mouse with muscle specific ablation of autophagy, implying association of accumulation of misfolded proteins and dysfunction or arrest of the autophagic process [4]. We have also found that APP, Aβ1-42, which are characteristic markers in DMRV and sIBM, as well as polyUb proteins and p62 [27]–[28], were accumulated in RV positive fibers. The chronic state of dysfunction or arrest of the autophagic process may secondarily cause the accumulation of amyloid proteins with long time. Interestingly, the myofibrils in the vicinity of the accumulated autophagic vacuoles maintained to be intact (Figure 4) as in those in DMRV and lysosomal myopathies [1], [11]. This finding may suggest the accumulation of autophagic vacuoles is independent of contractile machinery and the accumulated proteins would not be derived from the disrupted myofibrils.

## Supporting Information

Figure S1 **Immunohistochemical staining of macrophage marker and secondary antibody control in RV positive fivers in BMD compared to DMRV and sIBM.** Representative transverse serial sections of biopsied skeletal muscles from BMD with RV (left column), DMRV (center column) and sIBM (right column) patients. A–C: mGT staining. D–F: CD68, macrophage marker (red) co-stained with caveolin-3 (green), G–I: Alexa-labeled anti-mouse IgG secondary antibody (red) co-stained with caveolin-3 (green). Scale bar: 25 µm.
(JPG)
