# Supplementary figures and images for: Rimmed Vacuoles in Becker Muscular Dystrophy Have Similar Features with Inclusion Myopathies
Source: PLoS One. 2012 Dec 14;7(12):e52002. doi: 10.1371/journal.pone.0052002 (PMC3522649; doi:10.1371/journal.pone.0052002)

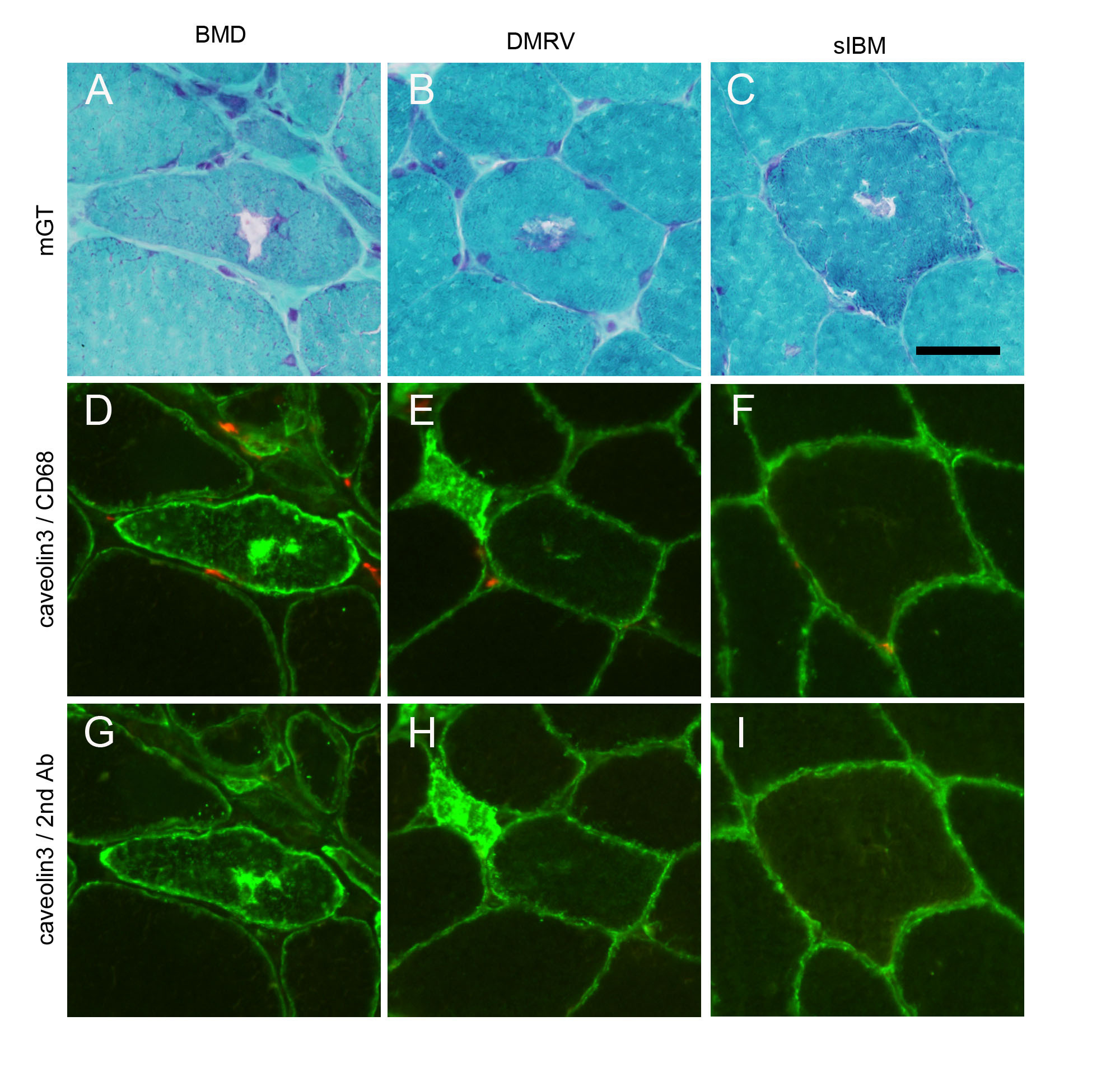

Supplement: Figure S1 — Immunohistochemical staining of macrophage marker and secondary antibody control in RV positive fivers in BMD compared to DMRV and sIBM. Representative transverse serial sections of biopsied skeletal muscles from BMD with RV (left column), DMRV (center column) and sIBM (right column) patients. A–C: mGT staining. D–F: CD68, macrophage marker (red) co-stained with caveolin-3 (green), G–I: Alexa-labeled anti-mouse IgG secondary antibody (red) co-stained with caveolin-3 (green). Scale bar: 25 µm. (JPG) [file pone.0052002.s001.jpg]
